# A Systematic Scoping Review on Migrant Health Coverage in Thailand

**DOI:** 10.3390/tropicalmed7080166

**Published:** 2022-08-03

**Authors:** Andrea König, Jamila Nabieva, Amin Manssouri, Khatia Antia, Peter Dambach, Andreas Deckert, Olaf Horstick, Stefan Kohler, Volker Winkler

**Affiliations:** 1 Heidelberg Institute of Global Health, Heidelberg University, 69120 Heidelberg, Germany; andrea.koenig@uni-heidelberg.de (A.K.); jamila.nabieva@uni-heidelberg.de (J.N.); khatia.antia@uni-heidelberg.de (K.A.); peter.dambach@uni-heidelberg.de (P.D.); a.deckert@uni-heidelberg.de (A.D.); olafhorstick@uni-heidelberg.de (O.H.); stefan.kohler@uni-heidelberg.de (S.K.); 2 Emergency Medicine Resident, Hôpitaux Universitaires de Genève, 1205 Geneva, Switzerland; amin.manssouri@gmail.com

**Keywords:** migrant health, universal health coverage, access to health care, Thailand, ASEAN, Southeast Asia

## Abstract

(1) Background: Thailand is a major migrant receiving country and pioneer of migrant health policy in the ASEAN region. However, on the ground, coverage of migrants is faced with multiple barriers. (2) Objectives: We aim to scope and analyse the types of available evidence on migrant health coverage in Thailand and identify knowledge gaps. Specifically, we characterise the literature along year of publication, migrant subpopulation, health domain, scope of coverage, methods, study design, objectives and results. (3) Methods: We searched PubMed, Web of Science, Google Scholar, Cochrane Database, Worldwide Science and the Asian Citation Index for peer-reviewed and grey literature in October 2021 for studies analysing original data on health coverage of migrants in Thailand. To conceptualise health coverage, we used the three dimensions availability, accessibility and acceptability. (4) Results: 101 articles were included in the final analysis. Sixty-three were published after 2016, 39 focused on migrant workers and 18 on migrants in general. Forty-two concentrated on health in broader terms, followed by reproductive and maternal health (*n* = 31). Thirty-eight assessed coverage of specific services and 36 health coverage in general. Migrants themselves and key informants were the main data sources in 80 and 43 of the articles, respectively. Forty publications were qualitative, while 38 applied quantitative methods (22% descriptive; 7% analytical). Among the health coverage components, 79 articles included aspects of accessibility, followed by acceptability (*n* = 59) and availability (*n* = 30). (5) Conclusions: While there is a high number and broad range of studies on migrant health coverage in Thailand, we found that research on migrant subgroups, such as victims of trafficking and migrant children, as well as on the health domains, non-communicable diseases and occupational and mental health is neglected.

## 1. Introduction

Southeast Asia is one of the regions with the highest population mobility in the world. Economic disparities and demographic differences lead to a high number of labour migrants [1]. Conflict and human trafficking further accelerate the trend in human mobility [2].

As an upper middle-income country with labour shortages, Thailand is a major recipient of migrant workers and hosts about 3.6 million workers from the bordering countries of Lao PDR, Cambodia and Myanmar (sometimes referred to as ‘CLM migrants’) [3]. Many migrant workers are employed in the informal sector in low-skilled so-called 3D jobs (dirty, dangerous and demeaning/demanding), such as agriculture, fisheries, domestic work, manufacturing and construction [4]. Although migrant workers are on average younger and healthier than the Thai host population, their health is negatively affected by social determinants, namely a higher risk of occupational injury and physical disability, stressful living and working conditions, but also adverse health behaviours. They are vulnerable to non-communicable diseases and mental health problems [5]. Moreover, infectious diseases, such as malaria, tuberculosis and HIV/AIDS, have been reported to be a health concern [5,6,7,8].

In Thailand, a high number of migrant workers are irregular, meaning that they stay or work without legal immigration documents [2,9]. Irregular migrants and victims of trafficking are particularly vulnerable to violence and exploitation, including sexual exploitation and violence [10], which can lead to injury, depression, sexually transmitted infections, and harmful alcohol and substance use [5]. In 2019, half of all migrant workers in Thailand were women [11]. Women migrants face additional challenges in accessing sexual and reproductive health services and are at a higher risk of maternal mortality [12].

While Malaysia and Singapore are still considering whether to include migrants into their health systems, Thailand is seen as a pioneer of migrant health coverage in the ASEAN region. The Thai government issued a number of migrant health policies, including a migrant health insurance scheme [1]. There are two mechanisms to migrant health coverage: The Compulsory Migrant Health Insurance (CMHI) covers all migrants, except those employed in the formal sector who are insured in the Social Security Scheme [13]. The CMHI includes a yearly compulsory health screening, and since 2013, the scheme has been expanded to migrant children up to the age of seven years [1,14]. Theoretically, since 2014 migrants are enrolled in the CMHI as part of a comprehensive registration measure, the ‘one stop service’, at which they also receive legal documentation through nationality verification. However, due to challenges with the service, only about a third of migrants in the country have enrolled [15]. Consequently, migrants face barriers in access to health care. Health seeking behaviour-related barriers are a lack of legal documents [1] and financial resources [1,2], language barriers [16], and lack of information [17]. Among the health system-related reasons are a frequent change of migration policies [2], the lack of a legal instrument enforcing enrolment in the migrant insurance [2], weaknesses in policy implementation [14], difficulties in managing the insurance among migrant employers, restriction of insurance benefits to the province [1] and upfront payment of fees for two years [1]. Moreover, providers are sometimes hesitant to enrol migrants due to pre-existing health conditions that threaten providers’ viability [15]. Consequently, migrants are prone to high out of pocket expenditures [1].

Researchers from various disciplines investigate health coverage through different methods and perspectives; hence, the literature is heterogeneous and lends itself to a scoping review approach. A 2015 scoping review on universal health coverage of migrants in the ASEAN region has shed light on different ASEAN members’ migrant health policies [1]. We aim to complement this review by scoping and analysing the types of available evidence on migrant health coverage in Thailand and identifying knowledge gaps [18]. For this purpose, we are focusing on migrant populations that are vulnerable to ill-health, which, as outlined above, includes migrant workers [19] and their dependents, victims of trafficking [20], refugees [21] and asylum seekers [22]. As it allows for a comprehensive overview of health coverage aspects, we used the dimensions of availability, accessibility and acceptability of the Tanahashi model for health service coverage to stratify our analysis. The Tanahashi model was designed to define and evaluate health service coverage from allocation of resources to achievement of the desired objective. It includes the components availability, accessibility, acceptability, contact and effectiveness coverage [23]. Specifically, we characterise the literature along year of publication, migrant subpopulation, health domain, scope of coverage, methods, study design, objectives and results.

## 2. Materials and Methods

We followed the methodological guidance for conducting systematic scoping reviews [24]. For reporting, we used the Preferred Reporting Items for Systematic Review and Meta-Analysis (PRISMA) extension for scoping reviews and the Joanna Briggs Institute Manual for Evidence Synthesis [18,25]. No protocol was submitted. To define health coverage, we used three dimensions—availability, accessibility and acceptability. Availability refers to the existence of health services for migrants, including resources such as facilities, workforce and availability of drugs. Accessibility contains factors determining migrants’ access to available health services, e.g., affordability, distance, freedom of movement, transport, legal documents, awareness and information, working arrangements and mobility. Acceptability includes factors such as language and cultural aspects, religion, discrimination, waiting time, confidentiality, trust and safety [23].

### 2.1. Search

To build the search syntax, we collected search terms for the elements ‘Population’, ‘Context’ and ‘Concept’ from literature (Supplementary File S1) [24]. Synonyms, UK/US terminology, medical/lay terms, acronyms/abbreviations, as well as terms from the MeSH (Medical Subject Headings) index were added afterwards. We searched PubMed (Supplementary File S2), Web of Science, Google Scholar, Cochrane Database and the Asian Citation Index for peer-reviewed and Worldwide Science and Google Scholar for grey literature with the latest search conducted on 6 October 2021. As grey literature, we included non-peer reviewed research papers, such as theses or research published in organisation reports. Reference lists of included publications were screened for additional relevant articles.

### 2.2. Inclusion Criteria and Data Extraction

We included primary research articles on migrant groups that are vulnerable to ill-health (migrant workers and their dependents, victims of trafficking, refugees and asylum-seekers) living in Thailand and that are examining at least one of the three health coverage dimensions (availability, accessibility and acceptability) (see Supplementary File S1). Articles missing any of the key elements of a scientific publication, such as methods or references, were excluded. After deletion of duplicates, two reviewers conducted title and abstract screening (AK and JN for peer-reviewed literature, AK and AM for grey literature). AK and VW conducted full article screening independently. Disagreements were solved by discussion. Data were mapped according to a ‘descriptive-analytical’ approach; we applied a common analytical framework and collected standard information from each study [26]. Extracted data items included health coverage dimension, year of publication, type of migrant population, health domain, scope of health coverage, methods broad, study design, methods detailed and perspectives represented. A glossary of these items is available in Supplementary File S3. Authors often used the general term ‘migrants’, when actually it was clear from the text that the population of interest were migrant workers. In such cases, we coded the population as migrant workers. For study designs, we assigned as described in the respective article. If the applied study design was not named, we assigned a category according to the described methodology. Studies analysing secondary data were only coded as secondary data analysis, including cross-sectional and longitudinal study designs.

We created figures for all data items stratified by health coverage dimension and conducted a descriptive qualitative content analysis of the objectives and results of included publications, aiming to provide an overview of included articles [18]. Classifications for objectives were developed inductively from the data, while results were sorted under the three health coverage dimensions deductively.

## 3. Results

### 3.1. Study Selection

**Figure 1:**
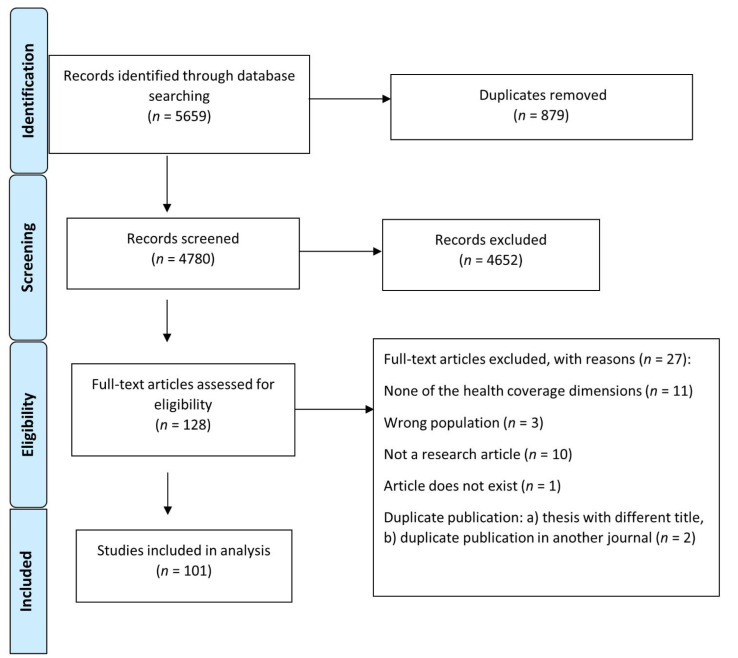
PRISMA flow diagram. Numbers of sources of evidence screened, assessed for eligibility and included in the review.

After removal of duplicates, we screened 4780 papers for title and abstract. Subsequently, we assessed 128 full articles, and after applying inclusion and exclusion criteria, 27 were excluded. Eleven papers were identified by screening reference lists. Finally, 101 studies were included (Figure 1), 96 of which were peer-reviewed and 5 grey literature articles. Ninety-nine articles were in English and two in Thai. Supplementary File S4 displays the data items for each paper.

### 3.2. Characteristics of Studies

In this section, we describe the frequency of data item categories for each data item. As many articles include more than one category, percentages often do not add up to 100%.

**Figure 2:**
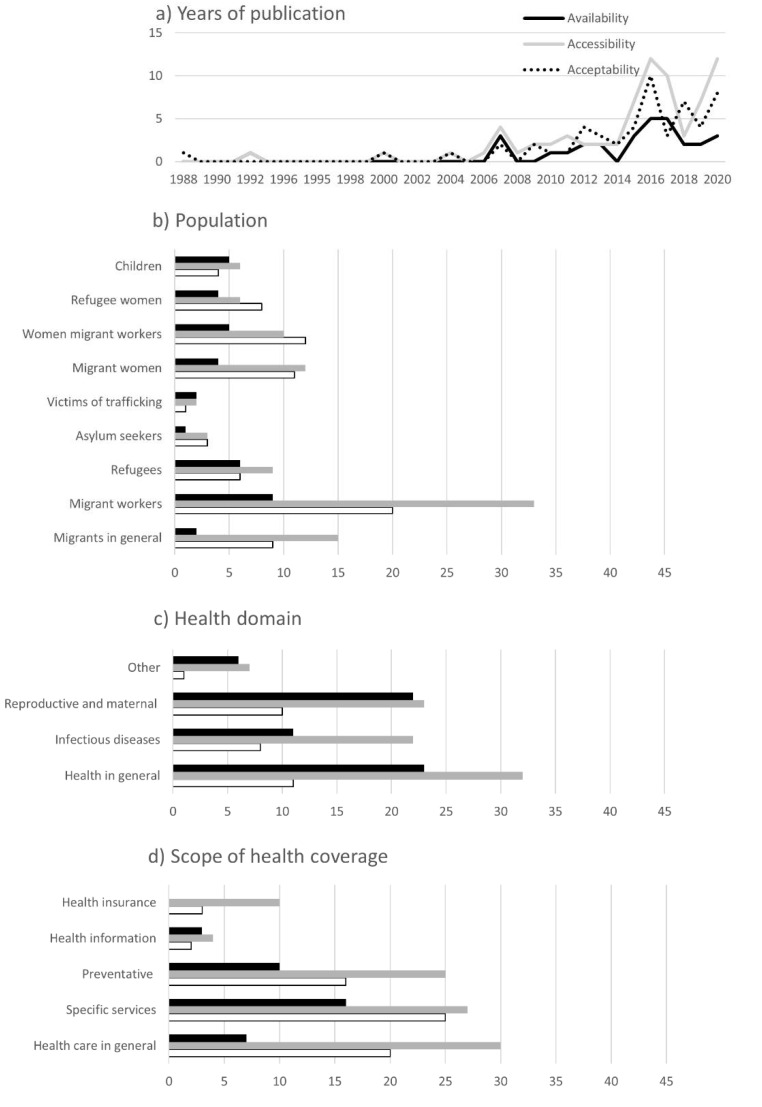
Charts of synthesised data items, including (**a**) years of publication (*x*-axis = year, y-axis = number of articles), (**b**) type of migrant population, (**c**) health domain and (**d**) scope of coverage. (*x*-axis = number of articles, availability = black, accessibility = grey, acceptability = white).

Thirty-eight percent of articles were published between 1975 and 2015 (Figure 2a). The number increased thereafter, with 63% of manuscripts published between 2016 and 2021. The distribution of health coverage dimensions remained similar throughout the years.

Thirty-nine percent of studies included migrant workers, followed by migrants in general (18%), refugees (15%)—asylum-seekers were represented in 4% of studies—migrant women (16%) and women migrant workers (14%) (Figure 2b). Nine percent of studies dealt with refugee women and 9% with migrant children; 2% addressed victims of trafficking. A few studies explicitly stated that they included undocumented migrants as well [2,7,14,27,28,29,30]. Among studies on female populations, the acceptability dimension was more prevalent than among other populations. Studies on refugees, victims of trafficking and children more frequently featured the availability dimension than others. 

Forty-two percent of articles focused on all health domains (Figure 2c), followed by reproductive and maternal health (31%) and infectious diseases (26%). The main subcategories among infectious disease were HIV/AIDS (10%) and tuberculosis (9%), while immunisation (5%), infectious diseases in general (1%) and malaria (1%) were less represented. Other health domains include occupational health (3%), non-communicable diseases (3%) and mental health (2%). In line with female migrant populations, the acceptability dimension was more prevalent in the reproductive and maternal health domain—which was limited to females.

Access to specific health services was assessed in 38% and access to health services in general in 36% of articles (Figure 2d). Under the latter category, we included any article focusing on one or more particular service, such as antiretroviral therapy or TB care. Preventative health care was included in 29% of studies, followed by health insurance (10%) and health promotion or information (5%). A high proportion of studies on specific services assessed their availability and acceptability.

**Figure 3:**
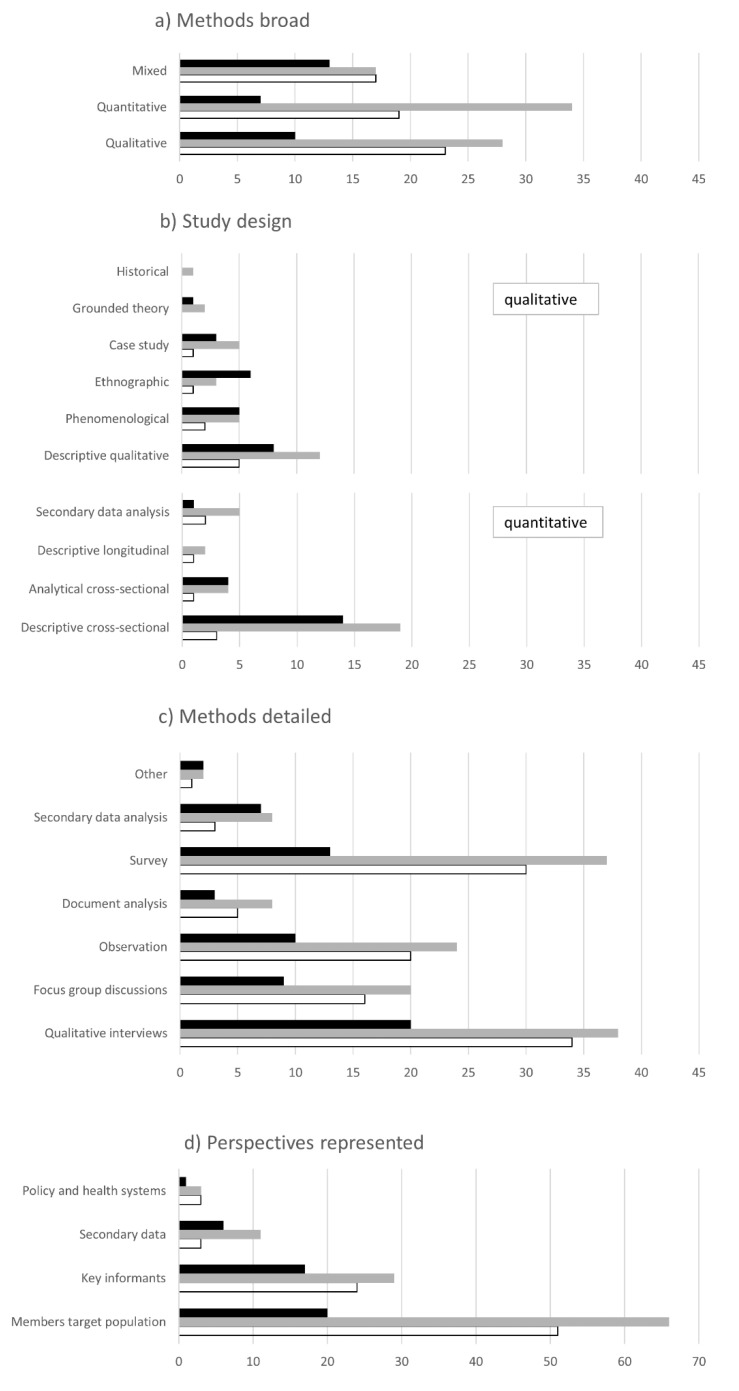
Charts of synthesised data items including (**a**) Methods broad, (**b**) Study design, (**c**) Methods detailed and (**d**) Perspectives represented. (Note: different scale than other diagrams). (*x*-axis = number of articles, availability = black, accessibility = grey, acceptability = white).

Qualitative methods were used in 40% of studies, 38% used quantitative and 23% mixed methods (Figure 3a). The sample sizes in qualitative approaches ranged from 7 to 165 and for quantitative from 72 to more than 70,000. Mixed-methods studies featured a high proportion of the availability dimension. Except for two longitudinal studies (2%), all quantitative studies were cross-sectional, with mainly descriptive designs (22%) and fewer analytical designs (7%) (Figure 3b). Six percent analysed secondary data, such as hospital patient records and data from health information systems. Among qualitative studies, many were descriptive qualitative (17%), followed by phenomenological (7%), ethnographic (7%) and case study (6%) designs. Only 2% used a grounded theory and 1% a historical design. Mixed methods studies were not coded for study design. Ethnographic, phenomenological and analytical cross-sectional studies focused mainly on the acceptability dimension. Analysis of secondary data was used frequently in studies assessing availability. Qualitative interviews (52%) and surveys (46%) were the most prominent methods (Figure 3c), followed by focus group discussions (27%), observation (14%), health information system data analysis (14%), document analysis (10%) and others (3%). More studies utilising qualitative interviews focused on accessibility and acceptability. Many studies represented multiple perspectives (44%). Members of the target population were included in 79% of studies (Figure 3d). Key informants, such as government representatives and policy makers, healthcare professionals and program managers of non-governmental organisations, trade unions and academic institutions, were interviewed in 43%. Secondary data (11%) and policy or health system documents (5%) were employed rarely. The availability dimension was more frequently assessed in studies utilising secondary data and key informant interviews.

### 3.3. Objectives and Results of Included Publications

The objectives of publications were derived from content analysis and are displayed in Table 1.

**Figure 4:**
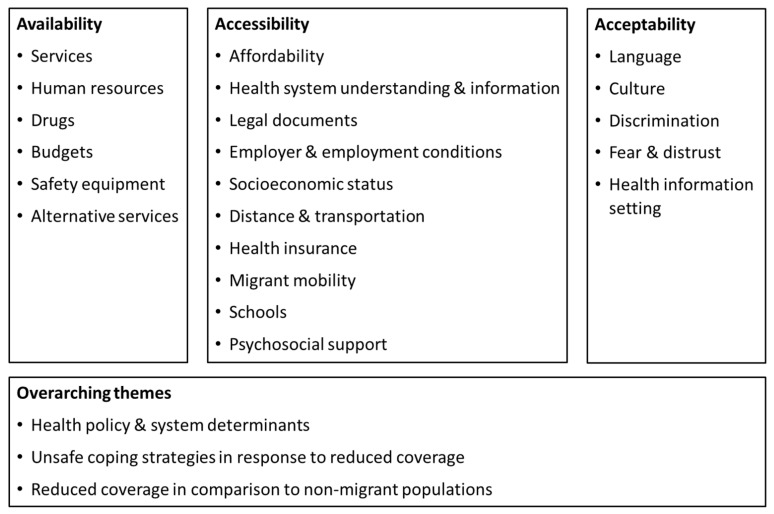
Themes developed from content analysis of results of individual sources of evidence, sorted against the three health coverage dimensions.

In Figure 4, we present an overview of the themes developed from content analysis of the results of included studies, sorted under the three health coverage dimensions. 

The following narrative synthesis is stratified by the health coverage dimensions and provides a detailed description of the themes shown in Figure 4.

#### 3.3.1. Availability

Availability was covered in few studies (Table 1, Figure 4). Some authors stated that budgets and human resources for migrant health were lacking [45,47,100]. Others revealed that specific health services, particularly sexual and reproductive health services and abortion, tuberculosis treatment, antiretroviral therapy or vaccinations for migrant children were not available or were limited for migrants [71,78,92,100] and relied heavily on donor funding [33,35]. One of these stated that only leftover vaccinations were offered to migrant children [92]. Furthermore, two articles stressed that occupational health care like protective equipment, care for injuries and employer support for work accidents, was lacking [10,101]. Three studies mentioned alternative service providers, such as a migrant clinic, informal migrant practitioners [95,102] and traditional healers [103].

#### 3.3.2. Accessibility

Many studies focused on barriers and some on contributors to accessibility from the perspective of migrants. One often cited barrier was affordability or high cost [42,43,49,55,65,69,77,81,83,104,105,106,107,108]. Accordingly, low-cost or free of charge services were identified as facilitators [13,60,82]. Studies also found that lack of health information or health system understanding as well as poor health-literacy reduced access to health care [17,27,31,39,44,55,66,67,68,78,80,81,83,84,91,95,105,109,110]. Complementary, information, staff communication, knowledge and perceived risk as well as school and health education were stated to increase access [16,51,69,100]. Eight publications described how lack of legal documents or documents being withheld by employers was associated with ineligibility to access services or fear of arrest [41,43,57,78,81,85,91,106]. Likewise, obtaining legal documents was cited as an enabling factor [42,69,82]. In some studies, the importance of documents also became apparent in the employment status. Only formally employed migrants were reportedly able to access health insurance [27,63]. Furthermore, the employer played an important role in hindering or facilitating access to health care. Authors stated that employers were resistant to enrol migrants in health insurance [29,40] and pay for care in case of injury [108]. In another study, they enabled access through supporting insurance enrolment [55]. Moreover, two studies found that migrants’ work responsibility and lack of rest days prevented access to care [83,106]. A few articles mentioned low socioeconomic status as a barrier [45,81,100]. Distance together with a lack of transportation to health care facilities reportedly reduced and proximity increased access [13,42,49,69,70,82,83,84,98,106,110]. Health insurance was popularly identified as a contributor and lack of it as an obstacle in access to health services [2,42,65,69,84,90,93,98]. Migrant mobility was stated to be a challenge in three studies, leading to loss of insurance benefits or loss to follow-up [7,27,85,106]. For children in particular, two studies found that schools enabled, sometimes partly, access to immunisation and other relevant health services [111,112]. Social or psycho-social support was cited as an access enabling factor [69,82,83,87].

#### 3.3.3. Acceptability

Challenges identified in many studies assessing acceptability were language [16,17,41,43,47,57,76,81,84,87,94,100,105,108,113] and cultural barriers [16,28,47,48,57,65,109]. Complementary, services in the migrant languages, providing interpreters and migrant health volunteers were identified as contributors [13,16,58,59,60,82]. Discrimination and stigmatisation of migrants and their health conditions were featured in eight studies [10,16,48,49,83,84,104,112]. Further reported deterrents included perceived lack of safety [13,60], confidentiality [61] and long waiting times [61,83,86,104,114]. Fear evolving from shyness and distrust was mentioned in a few studies and included fear of side effects, examinations, equipment, being seen in the hospital by acquaintances and being overcharged or misdiagnosed [83,84,110]. Three studies assessed preferred settings of receiving health information that made uncomfortable topics more acceptable. Those were group training about disease spread at the work place [76,115] or sexual and reproductive health information from friends, siblings, teachers and health workers for ‘very young adolescents’ [71].

Few studies addressed overarching themes. Some went beyond identifying barriers and focused on unsafe health seeking behaviours as a consequence of lacking health coverage. One study described how some migrants returned to their home country or did not seek treatment at all [7]. Unsafe behaviours included self-treatment, usage of unlicensed medications and self-induced abortion [7,42,43,57,78,85,86,89,99,110].

Studies comparing migrants’ health coverage to that of citizens found decreased coverage of migrants. One study revealed a lower usage of insurance and health care compared to ethnic minorities and Thais [88], and another stated that migrants self-treated more than Thais [43]. Migrants had a lower thalassemia awareness [105], pregnant migrant worker women used dental services less [109], and migrant children were worse off in comparison to Thai public school students regarding vaccinations and dental services [17,93]. The prevalence of unmet need among urban refugees and asylum-seekers was higher in both outpatient and inpatient services [28,40]. On the other hand, one study which compared access to care around a refugee camp compared to inside the camp, found that access inside the camp was easier and free of charge [107]. Another study revealed that, in one hospital, migrant Shan (ethnic group from Myanmar) women accessed antenatal care more than Thai women [30].

#### 3.3.4. Overarching Themes

Policy and health system challenges to migrant health coverage included limited corroboration and coordination among stakeholders and lack of migrant-inclusive data-systems [45,47,68,94,100,116]. Other reported shortcomings were conflicting policy directions and powerlessness of the ministry of public health [2]. Identified weaknesses of migrant health insurance were unclear policy messages, the voluntary nature, inadequate inter-ministerial coordination, bureaucratic hurdles, administrative delay and high costs rendering the insurance scheme ineffective [14,29,40,64,79].

## 4. Discussion

In Thailand, an increasing interest in migrant health coverage is apparent in the rising numbers of publications, especially over the past five years. Migrant workers and in particular migrant (worker) women are the focus of many studies, while other migrant groups, such as victims of trafficking and migrant children are still neglected. Moreover, studies on children of migrants almost exclusively focus on vaccination. Recently, some studies began to look at urban refugees and undocumented migrants revealing major barriers to health coverage. 

Health domains, such as infectious diseases and reproductive health, are frequently represented, whereas other relevant health domains, namely non-communicable diseases, occupational and mental health are lacking. The high burden of infectious diseases, such as HIV/AIDS and tuberculosis, is reflected in numerous articles on the respective service coverage. In contrast to the globally neglected topics of maternal and reproductive health among migrant workers [117], our review found that coverage of reproductive health services was well represented. With half of the migrant population in Thailand being women, the importance of this topic seems reflected [12]. Similar to the global level, there is a lack of research on access to non-communicable disease and occupational health services among migrants. Given the expected disease burden due to migrants’ precarious living and working conditions as well as their high risk of interrupted chronic disease treatment, research is needed [5,118]. Again, in contrast to the global research focus, mental health is hardly studied among migrants in Thailand despite the fact that migrants face many mental health challenges resulting from the demands of unfamiliar and difficult living and working environments [5].

Many studies have identified barriers to health coverage, such as low formal education and lack of information or understanding of the health system. However, research on facilitators to improve coverage as exemplified in a study investigating the role of bicultural health workers has hardly been done [119]. Furthermore, longitudinal studies on migrants’ health coverage at different stages of their migration process would add additional insights, such as whether and how certain determinants of coverage change over time. Other recent studies identify challenges in the health system and focus on the historical and political context of migrant health policies and their implementation [2,14]. Health system research involving the policy level might be useful to improve health coverage of migrants.

### Strengths and Limitations

Our systematic scoping review is a comprehensive characterisation of the research landscape on migrant health coverage in Thailand assessing a broad spectrum of data items and providing an overview of themes represented in studies’ objectives and results. However, there are some limitations. A literature search was conducted in English; hence, studies without at least an English abstract were not retrieved. The applied search syntax may not have identified all studies related to health coverage. The underlying concept of health coverage with its health domains may have influenced our results, and choosing a different theory or framework might lead to other insights. Lastly, many studies included multiple coverage dimensions which were coded independently, masking which one was dominant.

## 5. Conclusions

While there is a high number and broad range of studies on migrant health coverage in Thailand, we found that some migrant groups and health domains were not explored according to their relevance. To fill research gaps, we recommend scholars to direct their attention to marginalised sub-populations, such as victims of trafficking and children, and migrant-specific health domains, such as non-communicable diseases and occupational and mental health. Moreover, researchers should define and elaborate their conceptual approaches to health coverage. This review informs scholars in their selection of research priorities and directs attention to less researched subgroups and health domains. Moreover, decision makers are provided with an overview of relevant knowledge resources and a description of supply and demand side challenges as well as successful approaches to migrant health coverage. 

## Figures and Tables

**Table 1:** Objectives of included studies—Themes derived from content analysis.

Theme	Description
Availability	Studies [31,32,33] aimed to identify available health services for certain migrant populations.
Migrant health needs	Study [34] stated to assess migrant health or service needs.
Health system responsiveness	Publications [28,35,36,37,38,39,40] went further to investigate health system functioning for migrants or responsiveness to health needs. Studies [17,41,42,43,44] aimed to explore health-seeking behaviours or preferences of migrants.
Health system stakeholder perspective	The focus of studies [2,16,45,46,47,48] was directed on the state, policy maker or provider perspective on challenges to migrant health coverage
Target population perspective	Studies [7,13,30,48,49,50,51,52,53,54,55,56,57,58,59,60,61,62] chose to examine target population perspectives.
Policy impact	Studies [2,14,33,63] looked at the effects of a new policy, policy reform, policy gaps or policy implementation on migrants.
Determinants, barriers and facilitators to health coverage	Studies [13,29,40,64,65,66,67,68,69,70,71,72,73,74,75,76,77] explored determinants of health coverage openly, whereas the authors of studies [27,31,44,49,51,78,79,80] directly aimed to identify barriers and/or facilitators and studies [66,81,82,83,84] coping strategies related to coverage.
Coping strategies related to limited health coverage	Referring to barriers, studies [57,85,86] focused on unsafe coping strategies or problematic use of services.
Role of specific factors for health coverage	Specific factors assumed to determine coverage, such as insurance, social support, bicultural translators, occupation, geographic factors or the human rights situation were focused on in articles [10,58,70,87,88,89,90].
Health coverage in comparison to other populations	Studies [40,63,65,91,92] went further to detect in-between group differences, such as between migrants and Thai, ethnic minority or stateless populations.
Evaluate migrant health intervention	The goal of studies [2,54,93,94,95,96,97,98,99] was to evaluate a certain health intervention, such as migrant health insurance or health promotion or a community-based programme to determine the impact or feasibility of the intervention.

## Data Availability

Data analysed in a scoping review are secondary data, either openly accessible or accessible through university libraries. The authors of this review do not have the right to openly make non-open access journal articles public.

## Supplementary Materials

The following supporting information can be downloaded at: https://www.mdpi.com/article/10.3390/tropicalmed7080166/s1, File S1: Table. Population, Context and Concept; File S2: Figure. Search Syntax PubMed; File S3: Figure. Glossary of data items; File S4: Table. Charting table.
