# Supplementary material for: A Systematic Scoping Review on Migrant Health Coverage in Thailand
Source: Trop Med Infect Dis. 2022 Aug 3;7(8):166. doi: 10.3390/tropicalmed7080166 (PMC9415742; doi:10.3390/tropicalmed7080166)
Supplement: Supplementary file 1 [file tropicalmed-07-00166-s001.zip › Supplementary File S1.pdf]

POPULATION	CONTEXT	CONCEPT
Migrants, particularly including labour migrants, migrant women, migrant children, seasonal migrants, victims of trafficking, refugees, asylum-seekers and stateless migrants	Thailand, Brunei, Burma/Myanmar, Cambodia, Timor-Leste, Indonesia, Laos, Malaysia, the Philippines, Singapore, and Vietnam, Southeast Asia, Greater Mekong Subregion, Indochina	Availability, accessibility, acceptability, utilization, coverage, uptake, affordability, delivery, provision of health care/ medical care, health services, health insurance, health system responsiveness, health equity
