# Supplementary material for: A Systematic Scoping Review on Migrant Health Coverage in Thailand
Source: Trop Med Infect Dis. 2022 Aug 3;7(8):166. doi: 10.3390/tropicalmed7080166 (PMC9415742; doi:10.3390/tropicalmed7080166)
Supplement: Supplementary file 1 [file tropicalmed-07-00166-s001.zip › Supplementary File S2.pdf]

(migrant* OR migrat* OR asylum* OR refugee* OR transient* OR immigrant* OR immigrat* OR emigrant* OR emigrat* OR foreign* OR alien* OR non-citizen* OR noncitizen* OR non-national* OR “non national*” OR non-resident* OR “non resident*” OR squatter* OR “cross-border population*” OR “mobile population*” OR ((seasonal OR temporary OR irregular OR illegal OR undocumented OR guest) AND worker*) OR stateless OR state-less OR nomad OR gypsy OR gypsies OR citizenship OR displaced OR trafficking OR “unaccompanied minor*” OR “temporary resident*” OR “long-term resident*” OR “cultural minorit*” OR “ethnic minorit*” OR “visa overstayer*” OR “recent entrant*” OR “new entrant*” OR “returned national*” OR outsider* OR “enslaved person*” OR slave* OR “indefinite resident*” OR “Transients and Migrants” [mesh] OR Refugees [mesh] OR “Emigrants and Immigrants” [mesh] OR “Human Trafficking” [mesh] OR “Enslaved Persons” [mesh]) AND (((“Health care” OR Healthcare OR health-care OR “medical care” OR “medical service*” OR “health service*” OR insurance* OR “care service*”) AND (utilization OR utilisation OR delivery OR access* OR coverage OR accept* OR uptake OR availab* OR affordab* OR provision* OR challenge* OR barrier*)) OR “health system response*” OR “health coverage*” OR “health insurance*” OR “health need*” OR “health equity” OR “delivery of health care” [Mesh] OR "Patient Acceptance of Health Care"[Mesh] OR "Insurance coverage"[Mesh] OR “Insurance, Health” [mesh]) AND (“South East* Asia*” OR “Southeast* Asia*” OR “South-east* Asia*” OR Mekong* OR ASEAN OR “Association of Southeast Asian Nations” OR Thai* OR Myanma* OR Burm* OR Brunei* OR Indonesia* OR Singapore* OR Vietnam* OR “Viet Nam*” OR Cambodia* OR Laos* OR “Lao people’s democratic republic*” OR Laotian* OR Lao OR “East Timor*” OR Timor-Leste* OR Malay* OR Philippin* OR filipin* OR Yunnan* OR “Guangxi Zhuang*” OR “Southeast Asia” [Mesh])
