# Supplementary material for: A Systematic Scoping Review on Migrant Health Coverage in Thailand
Source: Trop Med Infect Dis. 2022 Aug 3;7(8):166. doi: 10.3390/tropicalmed7080166 (PMC9415742; doi:10.3390/tropicalmed7080166)
Supplement: Supplementary file 1 [file tropicalmed-07-00166-s001.zip › Supplementary File S3.pdf]

DATA ITEM	DESCRIPTION
health coverage dimension	Dimension from the Tanahashi health coverage model
year of publication	Year in which the paper was published
type of migrant population	Typology of migrant population according to definition or clarification in paper
health domain	Different areas of health and disease according to medical practice, which partly overlap
scope of health coverage	Fields or types of health care services
methods broad	Broad distinction of research approach according to qualitative, quantitative or a mix of both
study design	framework or the set of methods and procedures used to collect and analyse data
methods detailed	principles and procedures used for data generation
perspectives represented	Perspectives represented as sources of data analysed in the study
