# Supplementary material for: A Systematic Scoping Review on Migrant Health Coverage in Thailand
Source: Trop Med Infect Dis. 2022 Aug 3;7(8):166. doi: 10.3390/tropicalmed7080166 (PMC9415742; doi:10.3390/tropicalmed7080166)
Supplement: Supplementary file 1 [file tropicalmed-07-00166-s001.zip › Supplementary File S4.pdf]

REFERENCE	HEALTH COVERAGE DIMENSION	YEAR OF PUBLIC ATION	TYPE OF MIGRANT POPULATION	HEALTH DOMAIN	SCOPE OF HEALTH COVERAGE	METHODS BROAD	STUDY DESIGN	METHODS DETAILED	PERSPECTIVE REPRESENTED	N
Phanwichatkul et al.	acceptability	2021	migrant women	reproductive and maternal health	access to specific health services	qualitative	ethnographic	in-depth interviews and observation	migrants and key informants	13 migrant women and 10 key informants
Aung and Panza	accessibility and acceptability	2014	migrants	TB (infectious diseases)	access to specific health services	quantitative	descriptive cross-sectional	survey	migrants	392
Steinbrook et al.	accessibility	2021	migrant women	reproductive and maternal health	access to specific health services	quantitative	secondary data analysis	health information system	health information system	11624
Yimyam	acceptability	2014	migrant worker women	reproductive and maternal health and general	access to specific health services	mixed methods	mixed methods	survey and FGDs	migrants and key informants	314 survey + 20 in-depth interviews + 12 KIIs + 48 FGD participants
Kosiyaporn et al.	availability and accessibility and acceptability	2018	migrant children	immunization (Infectious diseases)	preventative health care	mixed methods	mixed methods	qualitative interviews and health information system	migrants and health information system	
Traisuwan	accessibility and acceptability	2021	migrant worker women	dental health	access to specific	quantitative	analytical cross-sectional	questionnaire	migrants	418

REFERENCE	HEALTH COVERAGE DIMENSION	YEAR OF PUBLICATION	TYPE OF MIGRANT POPULATION	HEALTH DOMAIN	SCOPE OF HEALTH COVERAGE	METHODS BROAD	STUDY DESIGN	METHODS DETAILED	PERSPECTIVE REPRESENTED	N
					health services					
Vittaporn and Boonmongkon	availability and accessibility and acceptability	2016	migrant workers	TB (Infectious diseases)	access to specific health services	qualitative	ethnographic	observation, in-depth interviews, field notes, and formal and informal interviews	migrants and key informants	43
Aiko et al.	accessibility	2016	migrant children	immunization (Infectious diseases)	preventative health care	quantitative	longitudinal descriptive	health information system	health information system	12277
Xu et al.	accessibility and acceptability	2021	migrant workers	NCDs (thalassemia)	preventative health care	quantitative	analytical cross-sectional	survey	migrants	492
Phaiyarom et al.	accessibility and acceptability	2021	urban refugees and asylum-seekers	NCDs	access to specific health services	quantitative	analytical cross-sectional	questionnaire	migrants	181
Kunpeuk et al.	accessibility and acceptability	2021	urban refugees and asylum-seekers	general	access to health care in general	quantitative	descriptive cross-sectional	survey	migrants	181
Pyae Phyo Kyaw and Geater	accessibility	2021	migrant workers (seafarers)	general	access to health care in general	quantitative	descriptive cross-sectional	questionnaire	migrants	385
Srisai et al.	accessibility	2020	migrant workers	general	health insurance	qualitative	case study	in-depth interviews	migrants and key informants	14
Kosiyaporn et al.	availability and accessibility and acceptability	2020	migrant workers	general	access to health care in general	qualitative	system analysis	In-depth interviews	key informants	50

REFERENCE	HEALTH COVERAGE DIMENSION	YEAR OF PUBLICATION	TYPE OF MIGRANT POPULATION	HEALTH DOMAIN	SCOPE OF HEALTH COVERAGE	METHODS BROAD	STUDY DESIGN	METHODS DETAILED	PERSPECTIVE REPRESENTED	N
Thein and Thepthien	accessibility	2020	migrant women	reproductive and maternal health	preventative health care	quantitative	descriptive cross-sectional	survey	migrants	360
Srivirojana et al.	accessibility	2014	migrant workers	general and occupational health	access to health care in general and preventative health care	qualitative	case study	in-depths interviews	key informants	60
Suphanchaimat et al. (a)	accessibility	2019	migrant workers	general	access to health care in general and health insurance	quantitative	secondary data analysis	health information system	health information system	1733 uninsured and 2122 insured
Prakunwisit and Areesantichai	accessibility	2015	migrant children	immunization (Infectious diseases)	preventative health care	quantitative	descriptive cross-sectional study	structured questionnaire	migrants	386
Chatchawanchanchanakij	availability and accessibility and acceptability	2007	migrant workers	general	access to health care in general	quantitative	descriptive cross-sectional study	questionnaire	migrants	300
Saether et al.	accessibility	2007	migrant workers	HIV/AIDS (Infectious diseases)	access to specific health services	qualitative	descriptive qualitative	FGDs and interviews	migrants and key informants	6 FGDs with 74 migrants and 13 KIIs
Arnott et al.	availability and accessibility and acceptability	2017	migrant women	reproductive and maternal health	access to specific health services	qualitative	descriptive qualitative	in-depth interviews	migrants	14

REFERENCE	HEALTH COVERAGE DIMENSION	YEAR OF PUBLIC ATION	TYPE OF MIGRANT POPULATION	HEALTH DOMAIN	SCOPE OF HEALTH COVERAGE	METHODS BROAD	STUDY DESIGN	METHODS DETAILED	PERSPECTIVE REPRESENTED	N
Myo-Lin-Zaw et al.	accessibility and acceptability	2016	migrant women	reproductive and maternal health	preventativ e health care	quantitativ e	descriptive cross-sectional study	structured questionnai re	migrants	402
Aung et al.	accessibility and acceptability	2009	migrant workers	general	access to health care in general	quantitativ e	descriptive cross-sectional study	structured questionnai re	migrants	388
Naing et al.	accessibility	2020	migrant workers	general	access to health care in general and health insurance	quantitativ e	descriptive cross-sectional study	survey	migrants	240
Isarabhakdi	accessibility and acceptability	2004	migrant workers	general	access to health care in general	mixed methods	mixed methods	survey and in-depth interviews and FGDs	migrants and key informants (health providers)	6656
Seo	acceptability	2016	migrants	general	access to health care in general (timeliness)	qualitative	ethnograph ic	in-depth interviews and observatio n	migrants	23
Toothong et al.	availability, accessibility and contact	2015	(irregular) migrant workers	infectious diseases	preventativ e health care	mixed methods	mixed methods	structured questionnai re and key informant interviews	migrants and key informants (health personnel)	767
Harris	accessibility	2013	migrant workers	general	access to health care in general	qualitative	historical	document analysis and interviews	policy/health system	120 KIIs

REFERENCE	HEALTH COVERAGE DIMENSION	YEAR OF PUBLICATION	TYPE OF MIGRANT POPULATION	HEALTH DOMAIN	SCOPE OF HEALTH COVERAGE	METHODS BROAD	STUDY DESIGN	METHODS DETAILED	PERSPECTIVE REPRESENTED	N
Canavati et al.	accessibility	2011	migrant worker children	immunization (Infectious diseases)	preventative health care	qualitative	descriptive qualitative	FGDs	migrants	53
Belton and Whittaker	availability and accessibility	2007	migrant worker women	reproductive and maternal health	access to specific health services and preventative health care	mixed methods	mixed methods	health information system and FGDs and in-depth interviews	migrants and key informants (health personnel) and health information system	232 patient records; 15 participant midwife group discussions; semi-structured interviews and conversations: women inpatients with post-abortion complications (43), their male partners' (10), health workers (20)
Belton	availability, accessibility, acceptability	2007	refugee women and migrant	reproductive and maternal health	preventative health care and	mixed methods	mixed methods	health information system	health information system,	180 medical case notes,

REFERENCE	HEALTH COVERAGE DIMENSION	YEAR OF PUBLIC ATION	TYPE OF MIGRANT POPULATION	HEALTH DOMAIN	SCOPE OF HEALTH COVERAGE	METHODS BROAD	STUDY DESIGN	METHODS DETAILED	PERSPECTIVE REPRESENTED	N
			worker women		access to specific health services			and semi-structured interviews and FGDs	migrants and key informants (health personnel)	31 referral records, 14 hospital case notes of deceased women, 43 in-depth interviews with women, 10 in-depth interviews with husbands, 20 interviews with lay midwives
Crozier et al.	accessibility and effective coverage	2008	migrant women	HIV/AIDS (Infectious diseases)	preventative health care	qualitative	grounded theory	in-depth interviews	migrants	38 migrant pregnant women; 26 health personal
Hegde et al.	availability and accessibility and acceptability	2011	migrant worker women	reproductive and maternal health	access to specific health services and preventative health care	mixed methods	mixed methods	survey and in-depth interviews and document analysis	migrants	15 surveys and 10 in-depth interviews, 15 KIIs

REFERENCE	HEALTH COVERAGE DIMENSION	YEAR OF PUBLIC ATION	TYPE OF MIGRANT POPULATION	HEALTH DOMAIN	SCOPE OF HEALTH COVERAGE	METHODS BROAD	STUDY DESIGN	METHODS DETAILED	PERSPECTIVE REPRESENTED	N
Hobstetter et al.	availability, accessibility and effective coverage	2015	migrant women and refugee women	reproductive and maternal health	access to specific health services	qualitative	descriptive qualitative	document analysis and interviews and FGDs and others (service mapping exercise)	migrants and key informants (organizations and health personnel)	46 key informant interviews with representa tives from 25 organizatio ns, 18 focus group discussions with migrant adults, migrant adolescent s, and healthcare workers, and a service mapping exercise with 22 stakeholde rs
Holumyong et al.	acceptability	2018	migrant worker women and migrant	reproductive and maternal health	access to specific health services and	quantitativ e	descriptive cross-sectional	survey	migrants	987

REFERENCE	HEALTH COVERAGE DIMENSION	YEAR OF PUBLICATION	TYPE OF MIGRANT POPULATION	HEALTH DOMAIN	SCOPE OF HEALTH COVERAGE	METHODS BROAD	STUDY DESIGN	METHODS DETAILED	PERSPECTIVE REPRESENTED	N
			worker children		preventative health care					
Hu	accessibility and contact coverage	2010	refugees and migrant workers	general	access to health care in general & health insurance	quantitative	secondary data analysis	health information system	health information system	?
Kågesten et al.	availability and accessibility	2017	refugee children	reproductive and maternal health	preventative health care & health promotion or information	quantitative	descriptive cross-sectional	survey	migrants	399
Kaji et al.	availability and effective coverage	2015	refugees and migrant workers	TB (Infectious diseases)	access to specific health services	qualitative	descriptive qualitative	in-depth interviews	key informants (health personnel, health policy makers)	31
Khongthanachayopit and Laohasiriwong	accessibility	2017	migrant workers	general	access to health care in general	quantitative	descriptive cross-sectional	structured questionnaire	migrants	621
Kunstadter	accessibility and acceptability	2013	migrants	HIV/AIDS (Infectious diseases)	health care in general	quantitative	analytical cross-sectional	survey	migrants	1169
Leiter et al.	accessibility	2006	migrant worker women	HIV/AIDS (Infectious diseases) and occupational	access to specific health services	qualitative	phenomenological	interviews	migrants and key informants	68 key in-formant interviews, 34

REFERENCE	HEALTH COVERAGE DIMENSION	YEAR OF PUBLIC ATION	TYPE OF MIGRANT POPULATION	HEALTH DOMAIN	SCOPE OF HEALTH COVERAGE	METHODS BROAD	STUDY DESIGN	METHODS DETAILED	PERSPECTIVE REPRESENTED	N
				health and RMNCH	and preventative health care					individual narratives
Maybin	availability and accessibility	1992	refugees	general	access to health care in general	mixed methods	mixed methods	health information system and observation and interviews	health information system, key informants (researchers), migrants	?
Murray et al.	accessibility and acceptability	2016	migrants	HIV/AIDS (Infectious diseases)	access to specific health services	qualitative	ethnographic and case study	observation and semi-structured interviews	migrants and key informants (health personnel)	16 interviews with patients, 7 KIIs
Musumari and Chamchan	accessibility and acceptability	2016	migrant workers	HIV/AIDS (Infectious diseases)	preventative health care	quantitative	descriptive cross-sectional	survey	migrants	1034
Naing et al.	accessibility, acceptability and contact coverage	2012	migrant workers	TB (Infectious diseases)	access to specific health services	quantitative	descriptive cross-sectional	survey	migrants	614
Phanwichatkul et al.	acceptability	2018	migrant women	reproductive and maternal health	access to specific health services (and health workforce)	qualitative	ethnographic	interviews and observation	migrants, key informants (including researchers)	4 Burmese interpreters, 9 health professionals, 10 Burmese women

REFERENCE	HEALTH COVERAGE DIMENSION	YEAR OF PUBLIC ATION	TYPE OF MIGRANT POPULATION	HEALTH DOMAIN	SCOPE OF HEALTH COVERAGE	METHODS BROAD	STUDY DESIGN	METHODS DETAILED	PERSPECTIVE REPRESENTED	N
Pocock et al.	acceptability	2020	migrant workers and refugees	general	access to health care in general	qualitative	descriptive qualitative	in-depth interviews and document analysis	policy/health system	44 Malaysia, 50 Thailand
Pocock et al.	availability or accessibility	2016	victims of trafficking	occupational health	preventative health care and and access to specific health services	quantitative	descriptive cross-sectional	survey	migrants	446
Pocock et al.	availability, accessibility and acceptability	2018	migrant workers and victims of trafficking	general	access to health care in general	mixed methods	mixed methods	survey and interviews	migrants and key informants	275 fishermen, 20 key informants
Pudpong et al.	accessibility	2019	(irregular) migrants	general	health insurance	qualitative	case study	interviews and FGDs and observation and document analysis	migrants and key informants (stakeholders)	20 interviews and 5 FGDs
Sirilak et al.	availability and acceptability	2013	migrant workers	general	healthcare in general (primary, health workforce)	mixed methods	mixed methods	in-depth interviews and observation and questionnaire	migrants and key informants (health personnel)	260 volunteers, 446 migrants

REFERENCE	HEALTH COVERAGE DIMENSION	YEAR OF PUBLICATION	TYPE OF MIGRANT POPULATION	HEALTH DOMAIN	SCOPE OF HEALTH COVERAGE	METHODS BROAD	STUDY DESIGN	METHODS DETAILED	PERSPECTIVE REPRESENTED	N
Srikanok et al.	availability, accessibility and contact coverage	2017	refugees	reproductive and maternal health	preventative health care	quantitative	longitudinal descriptive	health information system	health information system	not applicable
Sunpuwan et al.	accessibility and acceptability	2019	migrants and migrant workers	general	access to health care in general (drugs, timeliness)	qualitative	descriptive qualitative	in-depth interviews and FGDs and document analysis	migrants and key informants (drug suppliers and community members)	18 drug suppliers, 16 community members, 6 FGDs, inventories from 17 drug suppliers
Suphanchaimat et al. (b)	accessibility and acceptability	2019	irregular migrants and migrant workers	general	access to health care in general	qualitative	descriptive qualitative	in-depth interviews and legal document analysis	policy/health system	?
Suphanchaimat et al.	accessibility	2017	irregular migrants and migrant workers	general	access to health care in general	qualitative	descriptive qualitative	document analysis and in-depth interviews	policy/health system	7
Thetkathuek et al.	accessibility and contact coverage	2017	migrant workers	general	access to health care in general	mixed methods	mixed methods	survey and FGDs	migrants	861 participants in interviews /FGDs
Tousaw et al.	accessibility and acceptability	2017	migrant women	reproductive and maternal health	access to specific	qualitative	phenomenological	in-depth interviews	migrants	22

REFERENCE	HEALTH COVERAGE DIMENSION	YEAR OF PUBLIC ATION	TYPE OF MIGRANT POPULATION	HEALTH DOMAIN	SCOPE OF HEALTH COVERAGE	METHODS BROAD	STUDY DESIGN	METHODS DETAILED	PERSPECTIVE REPRESENTED	N
					health services					
Tschirhart et al.	accessibility and acceptability	2020	migrant women	reproductive and maternal health	access to specific health services	qualitative	descriptive qualitative	FGDs	migrants	72
Tschirhart et al. (a)	accessibility	2016	migrants and refugees	TB (Infectious diseases)	access to specific health services	qualitative	descriptive qualitative	interviews and FGDs	migrants and key informants (health personnel)	12 KIIs with public health officials and TB treatment providers; 11 FGDs with migrants and refugees
Tschirhart et al. (a)	accessibility	2017	migrants	TB (Infectious diseases)	access to specific health services	mixed methods	mixed methods	FGDs and interviews and survey	migrants and key informants	11 FGDs, 13 KIIs, 101 volunteers (survey)
Tschirhart et al. (b)	availability, accessibility	2016	migrants and refugees	TB (Infectious diseases)	access to specific health services	qualitative	phenomen ological	FGDs	migrants	61
Tschirhart et al. (b)	availability and effective coverage	2017	migrants	TB (Infectious diseases)	access to specific health services and	qualitative	descriptive qualitative	FGDs and interviews	key informants	13

REFERENCE	HEALTH COVERAGE DIMENSION	YEAR OF PUBLICATION	TYPE OF MIGRANT POPULATION	HEALTH DOMAIN	SCOPE OF HEALTH COVERAGE	METHODS BROAD	STUDY DESIGN	METHODS DETAILED	PERSPECTIVE REPRESENTED	N
					inclusion in health information systems					
Tuangratananon et al.	availability and acceptability	2019	migrant children	general	health promotion or information	qualitative	case study	interviews	key informants	34
Wangroongsarb et al.	accessibility and contact coverage	2011	(irregular) migrants and migrant workers	malaria (Infectious diseases)	access to health care in general	quantitative	analytical cross-sectional	survey	migrants	1800 (900 Myanmar, 900 Cambodian)
Webber et al.	availability and accessibility and acceptability	2012	migrant worker women	reproductive and maternal health	access to specific health services and preventative health care	mixed methods	mixed methods	FGDs & survey	migrants and key informants	18 beer promoter FGDs, 55 KII/groups
Webber et al.	accessibility and acceptability	2015	migrant worker women	reproductive and maternal health	access to specific health services and preventative health care	quantitative	descriptive cross-sectional	survey	migrants	390 beer promoters

REFERENCE	HEALTH COVERAGE DIMENSION	YEAR OF PUBLIC ATION	TYPE OF MIGRANT POPULATION	HEALTH DOMAIN	SCOPE OF HEALTH COVERAGE	METHODS BROAD	STUDY DESIGN	METHODS DETAILED	PERSPECTIVE REPRESENTED	N
Wongkongdech et al.	accessibility and acceptability	2015	migrant workers	TB (Infectious diseases)	access to specific health services & health insurance	quantitative	descriptive cross-sectional	structured questionnaire	migrants	4874
Titiporn et al.	availability and accessibility	2019	migrant children	general	health promotion or information and specific health services	quantitative	analytical cross-sectional	survey	migrants	375
Bodeker and Neumann	availability and acceptability	2012	refugees	general	access to specific health services	mixed methods	mixed methods	survey and in-depth interviews and FGDs and observation and document anaylsis and others	migrants and key informants (health personnel)	59 refugee and migrant surveys, 4 in-depth interviews with practitioners
Mon and Xenos	accessibility and acceptability and contact coverage	2015	migrant workers	general	health insurance	mixed methods	mixed methods	structured questionnaire and in-depth interviews and observation	migrants	?

REFERENCE	HEALTH COVERAGE DIMENSION	YEAR OF PUBLIC ATION	TYPE OF MIGRANT POPULATION	HEALTH DOMAIN	SCOPE OF HEALTH COVERAGE	METHODS BROAD	STUDY DESIGN	METHODS DETAILED	PERSPECTIVE REPRESENTED	N
Gedeon et al.	availability and accessibility and acceptability	2016	migrant women and refugee women	reproductive and maternal health	access to specific health services	qualitative	phenomen ological	interviews	migrants	31
Phanwichatk ul et al.	acceptability	2016	migrant women	reproductive and maternal health	access to specific health services (and health workforce) and preventativ e health care	qualitative	ethnograph ic	ethnograph ic interviews and observatio n	migrants and key informants (health personnel)	4 health profession als; 10 Burmese women
Boonchutim a et al.	accessibility	2017	migrants	HIV/AIDS (Infectious diseases)	health promotion or informatio n	quantitativ e	descriptive cross-sectional	questionnai re	migrants and key informants	106 PH officers
Nwi et al.	acceptability	2018	migrant workers	general	access to health care in general (and timeliness)	quantitativ e	descriptive cross-sectional	questionnai re	migrants	181
Srithongtha m et al.	availability and acceptability and effective coverage	2013	migrant workers	general	access to health care in general	mixed methods	mixed methods	FGDs and survey	policy/health system	2 FGDs/prov ince
Pinna et al.	availability and acceptability and contact coverage	2020	migrant children	immunization (Infectious diseases)	preventativ e health care	mixed methods	mixed methods	health informatio n system and FGDs	key informants (health personnel)	1707

REFERENCE	HEALTH COVERAGE DIMENSION	YEAR OF PUBLIC ATION	TYPE OF MIGRANT POPULATION	HEALTH DOMAIN	SCOPE OF HEALTH COVERAGE	METHODS BROAD	STUDY DESIGN	METHODS DETAILED	PERSPECTIVE REPRESENTED	N
Xu et al.	accessibility and acceptability	2019	migrants	NCDs	preventative health care	mixed methods	mixed methods	survey and interviews and FGDs	migrants and key informants (health personnel)	166 KAP survey, 9 in-depth-interviews, 3 FGDs
Kongvattananon et al.	accessibility and acceptability	2020	migrant worker women	reproductive and maternal health	access to health care in general	quantitative	descriptive cross-sectional	structured questionnaire	migrants	180
Kunpeuk et al.	accessibility	2020	(irregular) migrant workers	general	health insurance	qualitative	descriptive qualitative	others: group model building	key informants (stakeholders)	20
Boonchutima et al.	accessibility and acceptability	2020	migrant workers	HIV/AIDS (Infectious diseases)	health promotion or information	quantitative	descriptive cross-sectional	structured questionaire	migrants	386
Phaiyarom et al.	accessibility and contact coverage	2020	(irregular) migrants	general	access to health care in general and health insurance	quantitative	secondary data analysis	health information system	health information system	74.722
Kamonroek et al.	accessibility and contact coverage	2020	migrant workers	general	access to health care in general	quantitative	descriptive cross-sectional	survey	migrants	72
Kusakabe and Pearson	availability and accessibility and acceptability	2016	migrant worker women	general	access to health care in general	mixed methods	mixed methods	in-depth interviews and survey	migrants	165 in-depth interviews, 504 surveys, 20 follow-up interviews

REFERENCE	HEALTH COVERAGE DIMENSION	YEAR OF PUBLICATION	TYPE OF MIGRANT POPULATION	HEALTH DOMAIN	SCOPE OF HEALTH COVERAGE	METHODS BROAD	STUDY DESIGN	METHODS DETAILED	PERSPECTIVE REPRESENTED	N
Fellmeth et al.	acceptability	2018	migrant worker women and refugee women	mental health and reproductive and maternal health	access to specific health services	qualitative	phenomenological	in-depth interviews	migrants	11
Gonah et al.	accessibility and acceptability	2016	migrants	general	access to health care in general	qualitative	descriptive qualitative	FGDs	migrants	7 FGDs
Rijken et al.	acceptability	2012	refugee women and migrant worker women	reproductive and maternal health	preventative health care	mixed methods	mixed methods	survey and observation and interviews and FGDs	migrants and key informants (health personnel)	644
Morrison	accessibility and acceptability	2000	refugee women	reproductive and maternal health	preventative health care	mixed methods	mixed methods	interviews and FGDs and survey	migrants and key informants	102 women, 10 KIs, 28 men FGDs, 21 FGDs with TBAs
Cohen and Asgary	acceptability	2016	refugees and migrants	mental health	access to specific health services	qualitative	descriptive qualitative	FGDs and key informant interview	migrants and key informants	10 FGDs
Foster et al.	availability and accessibility and acceptability and effective coverage	2017	migrant women and refugee women	reproductive and maternal health	access to specific health services	quantitative	secondary data analysis	health information systems	health information system	918
Gedeon et al.	acceptability	2015	refugee women and migrant women	reproductive and maternal health	preventative health care	qualitative	phenomenological	in-depth interviews	migrants	31

REFERENCE	HEALTH COVERAGE DIMENSION	YEAR OF PUBLICATION	TYPE OF MIGRANT POPULATION	HEALTH DOMAIN	SCOPE OF HEALTH COVERAGE	METHODS BROAD	STUDY DESIGN	METHODS DETAILED	PERSPECTIVE REPRESENTED	N
Keller and Mongkolpuet	acceptability and effective coverage	1988	refugees	general	access to health care in general (health workforce)	quantitative	descriptive cross-sectional	survey	migrants	305
Seo	accessibility	2016	(irregular) migrant women	reproductive and maternal health	preventative health care	qualitative	ethnographic	observation and in-depth-interviews and health information systems	migrants and key informants	12 + other key informants
Tousaw et al.	accessibility and acceptability	2018	migrant worker women and refugee women	reproductive and maternal health	access to specific health services	qualitative	phenomenological	in-depth interviews	migrants	16
Legido-Quigley et al.	availability and acceptability	2020	refugees and asylum-seekers	general	access to health care in general	qualitative	descriptive qualitative	interviews	key informants	30
Suphanchaimat et al.	accessibility	2020	urban refugees/asylum-seekers	general	access to health care in general	quantitative	analytical cross-sectional	survey	migrants	181
Srisai et al.	accessibility and acceptability	2020	migrant workers	general	access to health care in general and health insurance	qualitative	case study	in-depth interviews	migrants and key informants	14
Brzezinski et al.	accessibility	2019	migrant workers	general and reproductive and maternal health	access to health care in general and	qualitative	descriptive qualitative	interviews	key informants and migrants	?

REFERENCE	HEALTH COVERAGE DIMENSION	YEAR OF PUBLICATION	TYPE OF MIGRANT POPULATION	HEALTH DOMAIN	SCOPE OF HEALTH COVERAGE	METHODS BROAD	STUDY DESIGN	METHODS DETAILED	PERSPECTIVE REPRESENTED	N
					preventative health care					
Jitthai et al.	availability	2010	migrant workers	HIV/AIDS (Infectious diseases)	access to specific health services	quantitative	secondary data analysis	document analysis and health information systems and survey and interviews	key informants	not applicable
Maskhao et al.	accessibility and contact coverage	2015	migrant workers	general	access to health care in general	mixed methods	mixed methods	interviews and observation and FGDs	migrants and key informants	30 FGDs, 6 KIIs
International Organization for Migration	accessibility and acceptability	2009	migrant workers and refugees	general	access to health care in general	mixed methods	mixed methods	document analysis and interviews and FGDs	migrants and key informants	not applicable
Chotiga	accessibility and acceptability?	2010	migrant women	HIV/AIDS (Infectious diseases) and reproductive and maternal health	preventative health services	qualitative	grounded theory	in-depth interviews and FGDs	migrants and key informants (health personnel)	38 Burmese pregnant women, 26 health care workers, 3 FGDs with HC workers
